# Estimation of the toxicity of sulfadiazine to *Daphnia magna* using negligible depletion hollow-fiber liquid-phase microextraction independent of ambient pH

**DOI:** 10.1038/srep39798

**Published:** 2016-12-22

**Authors:** Kailin Liu, Shiji Xu, Minghuan Zhang, Yahong Kou, Xiaomao Zhou, Kun Luo, Lifeng Hu, Xiangying Liu, Min Liu, Lianyang Bai

**Affiliations:** 1 College of Plant Protection, Hunan Agricultural University, Changsha 410128, PR China; 2 Biotechnology Research Center, Hunan Academy of Agricultural Sciences, Changsha 410125, PR China; 3 Southern Regional Collaborative Innovation Center for Grain and Oil Crops in China, Hunan Agricultural University, Changsha, PR China; 4 Collaborative Innovation Center of Farmland Weeds Control, Loudi, Hunan province, PR China

## Abstract

The toxicity of ionizable organic compounds to organisms depends on the pH, which therefore affects risk assessments of these compounds. However, there is not a direct chemical method to predict the toxicity of ionizable organic compounds. To determine whether hollow-fiber liquid-phase microextraction (HF-LPME) is applicable for this purpose, a three-phase HF-LPME was used to measure sulfadiazine and estimate its toxicity to *Daphnia magna* in solutions of different pH. The result indicated that the sulfadiazine concentrations measured by HF-LPME decreased with increasing pH, which is consistent with the decreased toxicity. The concentration immobilize 50% of the daphnids (EC50) in 48 h calculated from nominal concentrations increased from 11.93 to 273.5 mg L^−1^ as the pH increased from 6.0 to 8.5, and the coefficient of variation (CV) of the EC50 values reached 104.6%. When calculated from the concentrations measured by HF-LPME (pH 12 acceptor phase), the EC50 ranged from 223.4 to 394.6 mg L^−1^, and the CV decreased to 27.60%, suggesting that the concentrations measured by HF-LPME can be used to estimate the toxicity of sulfadiazine irrespective of the solution pH.

Approximately 50% of pre-registered organic compounds are ionizable. The categories of chemicals that have a greater tendency to be ionizable include pharmaceuticals and some classes of pesticides^1^,^2^. The dependence on pH of the toxicity and bioconcentration of ionizable organic compounds to organisms has been observed in many studies^3^,^4^,^5^,^6^. This dependence greatly influences the estimation of the toxicity and bioconcentration of ionizable organic compounds because the pH of natural waters fluctuates from 6 to 9^7^. Thus, risk assessment of ionizable pollutants in aquatic systems has been a great challenge^8^. Some researchers have advised using site-specific risk assessments for ionizable pharmaceuticals when making informed water management decisions^6^,^9^. Xing, *et al*.^10^ recommended that the water quality criteria for ionizable organic compounds should be determined as a function of pH.

Thus, a method to estimate the toxicity and bioconcentration of ionizable organic compounds that is independent on the environmental pH is urgently needed. Some models to predict the bioconcentration and toxicity of ionizable compounds based on pKa and the octanol-water partitioning coefficient, Kow or log P, have been developed^6^,^11^,^12^. However, there is no direct chemical method to predict the toxicity of ionizable organic compounds. The pH-dependent toxicity of ionizable organic compounds in organisms conforms to a toxicokinetic ion-trapping model^3^,^13^. The differing toxicities of ionizable organic compounds at different pH values can be attributed to the distinct permeabilities of the existing species (i.e., neutral and ionized forms) because neutral species can permeate biomembranes and become trapped in cells faster than the corresponding charged species, resulting in distinct differences in the internal concentrations. If the internal concentrations can be directly measured and used to calculate the toxicity, risk assessment could be improved irrespective of the environmental pH^14^. However, this determination is time-consuming and not suitable for risk assessment; therefore, optimizing a biomimetic method, such as three-phase hollow-fiber liquid-phase microextraction (HF-LPME), to estimate toxicity is important. In this method, the analytes of interest in aqueous samples pass through a thin layer (several microliters) of organic solvent immobilized within the pores of a porous hollow fiber and then pass into an acceptor solution inside the lumen of the hollow fiber^15^. We hypothesized that the concentration in the acceptor solution can be a surrogate for the internal effect concentrations. When the concentrations measured by three-phase HF-LPME are used to calculate the toxicities of ionizable organic compounds to organisms, the EC50 under different pH conditions should be the same, enabling estimation of the toxicity irrespective of the ambient pH.

Sulfonamide antibiotics are one of the most commonly prescribed groups of antibiotics globally in both human and veterinary medicine. These antibiotics are routinely detected in municipal wastewater effluent and surface waters in the low microgram-per-liter range^16^. The pKa, which describes the dissociation of the neutral form to the negatively charged form, of sulfadiazine is 6.5^17^, making the dissociation of sulfadiazine relevant in the environment, where even slight pH changes in the vicinity of the pKa will have a major impact on the balance between the neutral and ionized fractions (Fig. 1). Anskjær, *et al*.^5^ reported that the toxicity and bioconcentration of sulfadiazine in *Daphnia magna* depend on the pH. Hence, the objective of the present study was to use three-phase HF-LPME to measure sulfadiazine concentrations and estimate its toxicity and bioconcentration in *D. magna* in test solutions of different pH.

## Results and Discussion

### Effect of the test solution pH on the toxicity of sulfadiazine to *D. magna*

The pH in the test solutions was measured at 0, 24 and 48 h and found to be constant, with a maximum change of ± 0.28. *D. magna* grew well in all media at various pHs without sulfadiazine; no immobile animals were observed. The toxicity of sulfadiazine to *D. magna* decreased with increasing pH, the EC50 significantly increased with the pH, with values of 11.93, 97.28 and 273.51 mg L^−1^ at pH 6.0, 7.5 and 8.5, respectively (Table 1). The EC50 values at pH 7.5 and 8.5 were 9 and 22 times that at pH 6.0, respectively, and the coefficient of variation (CV) of the EC50 values at the three pH levels reached 104.6%. The 24-h toxicity decreased with increasing pH in the same manner as the 48-h toxicity (Table 1). Previous toxicity studies using standard procedures (pH 7.8 ± 0.2) indicated that the EC50 values (48 h) of sulfadiazine to for *D. magna* were 212–221 mg L^−1^ 18,19, which is between the values at pH 7.5 and pH 8.5; thus, our results were consistent with previous results reported by Anskjær, *et al*.^5^, with only the 48-h EC50 at pH 6.0 being slightly lower than the minimum limit (13.4 mg L^−1^). The toxicity decreased with increasing ionization at pH 8.5, where the sulfadiazine was almost completely ionized (99%), indicating that the neutral form was more toxic than the ionic form. Similarly, Xing, *et al*.^10^ found that the toxicities of weak organic acids, 2,4-dichlorophenol, 2,4,6-trichlorophenol and pentachlorophenol, to *D. magna* decreased with increasing pH, with significant correlations between the log-transformed acute toxicity (ln EC50/LC50) and pH. In the present study, because of the limits set by the pH tolerance and buffer sensitivity of *D. magna* and the use of only three pH levels, the correlations between the log-transformed acute toxicity (ln EC50/LC50) and pH could not be statistically analyzed. The pH-dependent aquatic toxicities of ionizable compounds have been of concern^3^,^4^,^5^,^6^,^10^, because these values affect risk assessment. In addition to the acute toxicity, the same total concentration of zwitterionic tetracycline in ambient solution can evoke very different expressions of the antibiotic resistance gene in the exposed bacteria due to differential antibiotic uptake at different pH values^20^. Therefore, a pH-independent method for the risk assessment of ionizable compounds is urgently needed.

### Effect of pH on the concentration of sulfadiazine detected by HF-LPME

**Figure 2:**
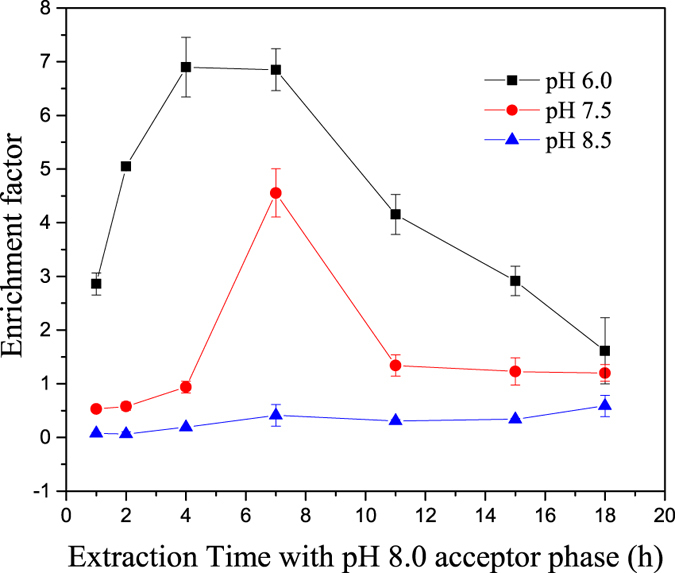
Influence of the extraction time on HF-LPME (pH 8.0 acceptor phase).

The extraction time was determined for sample solutions at pH 6.0, 7.5 and 8.5. The uptake profiles of sulfadiazine versus the extraction time are shown in Fig. 2, which indicates that the enrichment factor of sulfadiazine reached a maximum at 7 h (6.85, 4.55 and 0.44 for pH 6.0, 7.5 and 8.5, respectively) and then decreased slowly due to the loss in the supported liquid membrane^21^. Thus, the extraction time of 7 h was used in subsequent studies. HF-LPME with HPLC has been used to determine sulfonamides and their main metabolites^22^,^23^, but these studies often pursued the maximum enrichment factors; thus, the pH of the donor phase (sample) was adjusted to maintain the analytes in their non-ionized form without considering the different toxicities of the existing species. In the present study, the pH of the sample solutions remained constant in the *D. magna* toxicity tests. In HF-LPME applications, the sample is often stirred by a magnetic stirrer to speed up the extraction^21^,^24^. To directly analyze the environmental water *in situ*, a static HF-LPME was used in the present study, so the extraction time was longer.

**Figure 3:**
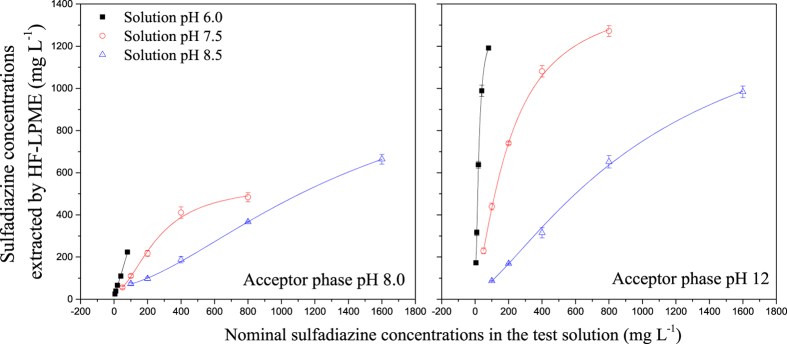
Sulfadiazine concentrations extracted using the three-phase HF-LPME (pH 8.0 and 12 acceptors) in pH 6.0, 7.5 and 8.5 test solutions.

The concentration of sulfadiazine in the acceptor phase decreased with increasing test solution pH when the acceptor phase pH was fixed, whereas the concentration of the pH 12 acceptor phase was significantly higher than that of the pH 8.0 acceptor phase (Fig. 3). In agreement with our results, previous studies have found that when the donor phase pH increased from 4.5 to 7.0, the enrichment factor significantly decreased^25^. With increases in the nominal concentrations in the test solutions, the rate of increase of sulfadiazine in the acceptor phase slows down. As shown in Fig. 3, when linear and logistic equations were used to fit the concentration increase in the acceptor phase against the nominal concentration, the logistic equation could be well fitted, with adjusted *R*^*2*^ (adj. *R*^*2*^) values ranging from 0.9887 to 0.9999 at all pH levels, compared to the linear equation, with adj. *R*^*2*^ values of 0.6631–0.9996. This finding indicates that the sulfadiazine concentration detected by HF-LPME increased with the nominal concentration according to a logistic model. Negligible depletion solid-phase microextraction coupled to high-performance liquid chromatography (nd-SPME-HPLC) has been used to quantify the free concentrations of ionizable antimicrobial compounds^26^,^27^. However, SPME applications for ionizable compounds have been limited because of the neutral charge on commercial SPME coatings, resulting in a low coating/sample partition coefficient and poor analyte recoveries^24^. Thus, HF-LPME with HPLC is more suited for the determination of ionizable compounds.

The sample depletion of the target compound was less than 5%, i.e., the criterion of negligible depletion^28^. In the present study, the maximum sample depletion was 4.15%, so the HF-LPME method was considered negligible depletion. The nd-HF-LPME has been used to detect freely dissolved triazine herbicide and phenol^29^,^30^. Both studies used two-phase HF-LPME; in the present study, three-phase HF-LPME was used to determine bioavailable sulfadiazine because this method is better suited for ionizable compounds, is particularly compatible with HPLC, and employs a similar extraction process to the toxicokinetic ion-trapping model.

### Estimating the toxicity of sulfadiazine to *D. magna* using the concentrations of sulfadiazine detected by HF-LPME at different pH levels

**Figure 4:**
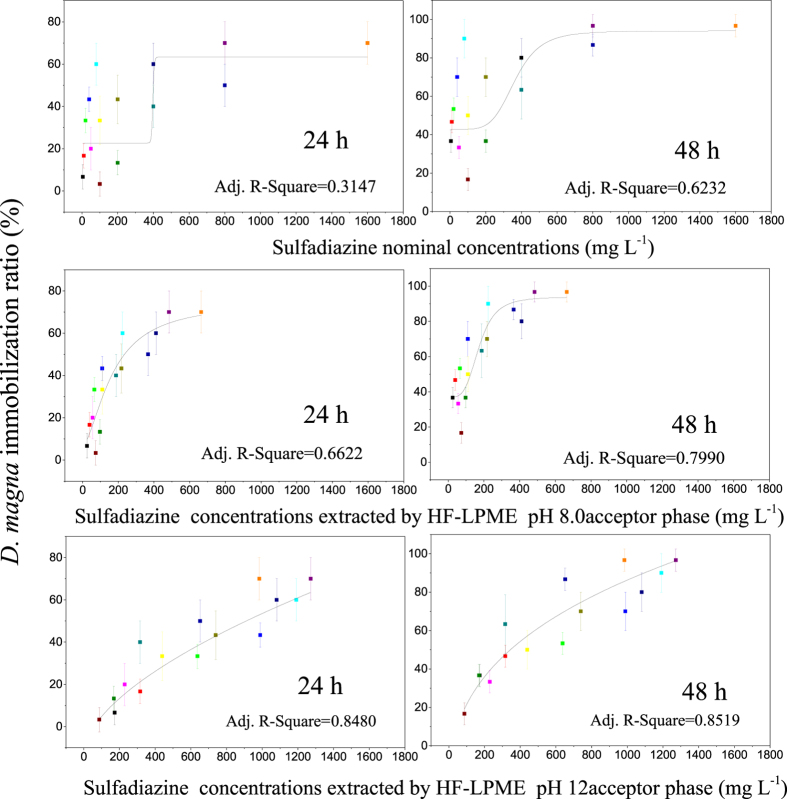
*D. magna* immobilization ratios at 24 and 48 h based on nominal sulfadiazine concentrations and sulfadiazine concentrations detected by HF-LPME (pH 8.0 and 12 acceptor phases).

In theory, if a measured concentration can be used to estimate the toxicity, the EC50 values for *D. magna* based on that concentration should be the same irrespective of the solution pH. The decreased CVs of the EC50 indicate the EC50 values were nearly the same. The EC50 values calculated from the sulfadiazine concentrations detected by HF-LPME at different pH levels are summarized in Table 1. Compared with the EC50 values calculated from the nominal concentrations, the variation in the EC50 values calculated from the sulfadiazine concentrations detected by HF-LPME at different pH values significantly decreased. For the EC50 calculated from the sulfadiazine concentrations detected by HF-LPME at an acceptor phase pH of 8.0, the CVs of the EC50 values at 24 h and 48 h decreased from 101.5–104.6% for the nominal concentration to 40.9–49.9%. When the acceptor phase pH was 12, the CV of the EC50 decreased to 27.9–30.7% (Table 1). These results indicated that the sulfadiazine concentrations detected by HF-LPME could improve the toxicity estimation for *D. magna* at different pH values. In theory, the pH 8.0 acceptor phase is similar to cellular pH, so the pH 8.0 acceptor phase can better estimate the toxicity. However, the pH 12 acceptor phase provided a better estimation than the pH 8.0 acceptor phase potentially because the enrichment did not reach equilibrium in the pH 8.0 acceptor phase and the enrichment factor decreased slowly only due to the loss in the supported liquid membrane^21^. In contrast, the enrichment in the pH 12 acceptor phase was more rapid than in the pH 8.0 acceptor phase, and equilibrium was achieved before loss of the supported liquid membrane. To study the feasibility of estimating the toxicity to *D. magna* using the sulfadiazine concentrations detected by HF-LPME, these concentrations and the immobilization ratio at all pH values were fit by a logistic model (Fig. 4). When the sulfadiazine concentrations detected by HF-LPME were used to fit the curve, the adj. *R*^2^ increased from 0.3147 for the nominal concentration to 0.8519 for the sulfadiazine concentrations detected by HF-LPME (acceptor pH 12). Due to the similarity of the increased immobilization ratio and the sulfadiazine concentrations detected by HF-LPME (pH 12 acceptor), the immobilization ratio and the sulfadiazine concentrations detected by HF-LPME (pH 12 acceptor) could also be well fitted by linear equations. The toxicity increased with the increase in the sulfadiazine concentration extracted by HF-LPME at different pH values, suggesting that the HF-LPME extracted concentration can be used to estimate the toxicity of sulfadiazine independent of the ambient pH, that was the concentrations of sulfadiazine in the test solutions measured by HF-LPME and the corresponding immobilization-concentration-response equation were used to calculate the immobilization ratio.

The mechanism estimating the toxicity of sulfadiazine based on HF-LPME presumes that the principle between HF-LPME detection and *D. magna* absorption of sulfadiazine is similar. In HF-LPME, the sulfadiazine in the solution passes through the organic liquid membrane (1-octanol) immobilized within the pores of a porous hollow fiber and then into an acceptor solution inside the lumen of the hollow fiber^15^, which is similar to the absorption by *D. magna* in a sulfadiazine solution. In *D. magna*, sulfadiazine permeates across biomembranes and becomes trapped inside the organism. In HF-LPME, the organic liquid membrane is used as a surrogate for the biomembrane, and the acceptor concentration is a surrogate for the internal concentration. Neutral species can pass through the organic liquid membrane and then into an acceptor solution faster than the corresponding charged species, similar to permeation across biomembranes and entrapment within cells. We attempted to determine the concentration of sulfadiazine inside *D. magna* organisms, but because the lower sample biomass (5-day-old *D. magna* with an average wet weight of 1.2 ± 0.2 mg; 20 daphnids weighed approx. 24 mg) and higher limit of quantification of HPLC, the concentration of sulfadiazine inside *D. magna* was not detected. However, in agreement with our detected concentrations by HF-LPME, previous measured whole-body concentrations of weakly acidic sulfonamides and weakly basic diphenhydramine in fish significantly increased with increases in the neutral molecule forms^31^,^32^, suggesting that the acceptor concentration in HF-LPME can be used as a surrogate for the internal concentration in *D. magna* to estimate the toxicity of sulfadiazine to *D. magna*. A detailed mechanism will be studied in the future.

Although HF-LPME improves the estimation of toxicity that is dependent on pH, the highest adj. R^2^ was only 0.8519; in other words, the estimation requires further improvement. In the present study, 1-octanol was used as the organic liquid membrane in the fiber pores in HF-LPME, which may not be an ideal biomembrane model. The liposome-water distribution ratio is a more suitable descriptor of the uptake of hydrophobic ionizable compounds into biological membranes than the corresponding octanol-water distribution ratio^33^. A hollow fiber filled with living cells or cell membranes coupled with HPLC, termed hollow-fiber cell fishing with HPLC (HFCF–HPLC), has been used to simulate the actual conditions of the interactions between active compounds and cells^34^,^35^. A hollow fiber with other similar biomembranes will be used to estimate bioavailability in a future study. In the present study, very little dissolved organic matter was present in the water, and all the sulfadiazine was freely dissolved. However, the toxicity of sulfadiazine depends on the solution pH. For ionizable organic compounds, the freely dissolved concentration cannot be used to predict the bioavailability. The speciation (i.e., neutral and ionized forms) must be considered because the neutral form is often easily absorbed by organisms^7^. Although SPME has been used to predict the toxicity or bioconcentration of hydrophobic organic contaminants in complex matrices, such as soil and sediment^36^,^37^, determining the ionizable compound content by SPME is difficult, and SPME cannot simulate the ion-trapping model. The three-phase HF-LPME approach is more often applied for the determination of ionizable (acidic or basic) compounds and may be the main tool used to predict the toxicity or bioconcentration of ionizable compounds in *D. magna* and other organisms in water and complex matrices in the future.

The toxicities of weak acids and weak bases to *D. magna* increase with an increasing neutral fraction^4^,^5^ and can be described by a toxicokinetic ion-trapping model^3^,^38^. Thus, in theory, HF-LPME can be used to estimate the toxicities of ionizable compounds. However, due to the added effect of electrostatic attraction, it is theoretically possible for the cation to be more toxic and more bioaccumulative. Estimation of the toxicity of a weak base by HF-LPME will be studied in the future.

## Conclusion

A negligible depletion three-phase HF-LPME method with HPLC was developed to detect sulfadiazine in water samples, and the detected concentrations decreased with increasing pH of the test solution. Similarly, the toxicity of sulfadiazine to *D. magna* also decreased with increasing test solution pH. When the concentrations detected by three-phase HF-LPME were used to fit the correlations between the concentration and immobilization ratio, the correlation significantly improved compared to that using the nominal concentration, independent of the solution pH, suggesting that three-phase HF-LPME is a useful tool for estimating the toxicity of sulfadiazine and perhaps other weakly acidic organic compounds, independent of the solution pH.

## Materials and Methods

### Chemicals and hollow fiber

All chemicals were of analytical reagent grade or better. Sulfadiazine (SDZ, 99.5%) was purchased from Dr. Ehrenstorfer GmbH (Augsburg, Germany). Q3/2 Accurel^®^ PP polypropylene microporous hollow fiber membrane (200-μm wall thickness, 600-μm inner diameter, 0.2-μm pore size, and 75% porosity) was obtained from Membrana GmbH (Wuppertal, Germany). All solutions and dilutions were prepared using ultrapure water from a Milli-Q Plus (Millipore, Billerica, MA, USA) water purification system.

### Acute toxicity testing

Toxicity tests were performed according to the performance criteria of OECD Guideline 202 (OECD, 2004), with the reconstituted water pH adjusted using the buffer recommendations of Rendal, *et al*.^39^, adding MES hydrate (9.2 mM) and tris (3.3 mM and 2 mM) to achieve stable pH levels of 6.0, 7.5 and 8.5, respectively. Sodium hydroxide (98%) and hydrochloric acid were used to adjust the pH of the buffer solutions. Based on preliminary bioassay tests (data not shown), 5 concentrations for each test solution −5, 10, 20, 40, and 80 mg/L for the pH 6.0 test solution; 50, 100, 200, 400, and 800 mg/L for pH 7.5; and 100, 200, 400, 800 and 1600 mg/L for pH 8.5 - and two control series with OECD M7 media and buffered media were tested. Each replicate consisted of a 100-mL glass beaker containing 50 mL of a test solution and 10 *D. magna* neonates (0–24 h). The test was placed in the dark at 20 ± 2 °C, and the number of immobilized animals was registered after 24 and 48 h. The pH was measured in the controls and at the highest concentrations. Twenty-five milliliters of the test solution was then extracted by three-phase HF-LPME. All of the treatments were performed in triplicate.

### Extraction procedure

The HF-LPME unit setup was similar to that described in a previous work by Tao, *et al*.^25^. Briefly, hollow fibers were cut into 12-cm pieces, washed with acetone in an ultrasonic bath and dried. The fiber was dipped in 1-octanol for 5 min to form an organic liquid membrane in the fiber pores. Before extraction, the surface and lumen of the fiber were flushed with water to remove the excess 1-octanol. The acceptor phase (pH 8.0 and 12.0 were determined by 3.3 mM TRIS and 0.01 M NaOH, respectively) was slowly injected into the fiber lumen using a disposable syringe until the lumen was full, and both open ends of the fiber were then folded and sealed with heated tweezers; the acceptor volume was approximately 30 μL. Next, the setup was immersed in 25 mL of a test solution in a 25-mL brown capped vial and allowed to sit. At the end of the extraction time, the fiber was carefully removed from the test solution, and both sealed ends were cut. One end was connected to the needle of a syringe full of air, and the acceptor phase containing sulfadiazine was then flushed out from the fiber lumen into a clean glass liner tube (250 μL, Alltech, Deerfield, IL, USA). Finally, 10 μL of the acceptor phase was injected into an HPLC system for analysis. To ensure that the extraction was free of memory effects and that the membrane life was not a concern, a new fiber was used for each extraction.

### Chromatographic conditions

Chromatographic separation was performed at 30 °C using an Agilent 1260 series liquid chromatography instrument (Palo Alto, CA, USA) equipped with a quaternary pump, vacuum degasser and thermostated column compartment. For detection, the HPLC was equipped with a series diode array (DAD). Separations were performed on a ZORBAX SB-C18 column (150 mm × 4.6 mm i.d.) with 5-μm particle size (Agilent Technologies, USA) preceded by a 5-μm ZORBAX SB-C18 (12.5 mm × 4.6 mm i.d.) analytical guard column, with a mobile phase of methanol and 0.2% acetic acid (25:75, v/v) at a flow rate of 0.8 mL/min. The extracted sulfadiazine sample (10 μL) was injected into the HPLC system, and the response was recorded at 265 nm, with a retention time of approximately 3.77 min for sulfadiazine.

### Statistics

The estimated concentrations to immobilize 50% of the daphnids within a stated exposure period (EC50) for the *D. magna* acute toxicity tests were derived by probit analysis using the program SPSS 22.0; the criterion of “non-overlapping 95% confidence intervals” was used to determine significant differences (p < 0.05) between the LC50 values^40^, and the CVs under different pH conditions were compared. Linear and logistic equations were used to fit the growth of the concentrations detected by HF-LPME to the nominal sulfadiazine concentrations.

## Additional Information

**How to cite this article**: Liu, K. *et al*. Estimation of the toxicity of sulfadiazine to *Daphnia magna* using negligible depletion hollow-fiber liquid-phase microextraction independent of ambient pH. *Sci. Rep.* **6**, 39798; doi: 10.1038/srep39798 (2016).

**Publisher's note:** Springer Nature remains neutral with regard to jurisdictional claims in published maps and institutional affiliations.

## Figures and Tables

**Figure 1:**
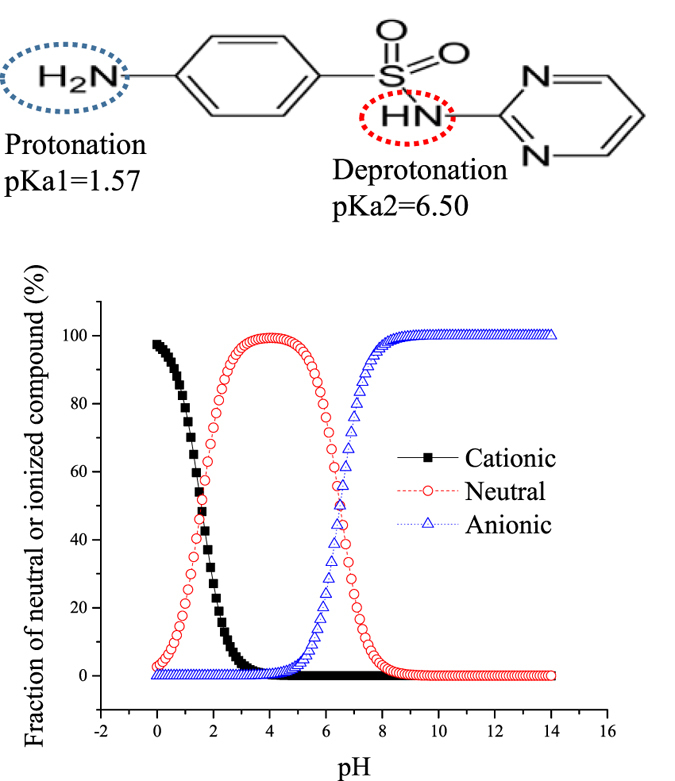
Sulfadiazine chemical structure and percent ionization at different pH^5^.

**Table 1:** EC50 values of sulfadiazine with 95% confidence intervals (CIs) for acute immobility tests with *Daphnia magna* at three different pH levels: 6.0, 7.5, and 8.5.

EC50 with 95% CI (mg L^−1^)	Time (h)	pH 6.0 (neutral = 76%)	pH 7.5 (neutral = 9%)	pH 8.5 (neutral = 1%)	CV (%)
EC50 based on nominal concentrations	24	49.89 (39.77–67.20)^a^	261.2 (206.9–339.4)^b^	749.5 (530.3–1217)^c^	101.5
	48	11.93 (4.832–20.28)^a^	97.28 (78.19–116.6)^b^	273.5 (238.4–311.4)^c^	104.6
EC50 based on concentrations detected by HF–LPME (pH 8.0 acceptor phase)	24	142.9 (118.9–182.1)^a^	242.9 (200.1–301.7)^b^	340.9 (229.9–647.9)^b^	40.87
	48	46.15 (37.54–54.79)^a^	104.6 (41.49–165.0)^ab^	143.4 (128.2–159.7)^b^	49.93
EC50 based on concentrations detected by HF–LPME (pH 12 acceptor phase)	24	1029 (876.9–1273)^a^	759.4 (653.5–898.2)^ab^	551.6 (398.8–874.6)^b^	30.69
	48	353.4 (0.8641–771.3)^a^	394.6 (176.2–572.6)^a^	223.4 (196.9– 251.6)^a^	27.60

Neutral indicates the fraction of undissociated compound; CV indicates the coefficient of variation of the EC50 values at different pH. Different letters in the EC50 line indicate significantly different values (p < 0.05) between the different pH levels.

## References

[b1] Franco A., Ferranti A., Davidsen C. & Trapp S. An unexpected challenge: ionizable compounds in the REACH chemical space. Int J Life Cycle Assess 15, 321–325 (2010).

[b2] Trapp S., Franco A. & Mackay D. Activity-Based Concept for Transport and Partitioning of Ionizing Organics. Environ. Sci. Technol. 44, 6123–6129 (2010). 20704208 10.1021/es100509x

[b3] Neuwoehner J. & Escher B. I. The pH-dependent toxicity of basic pharmaceuticals in the green algae Scenedesmus vacuolatus can be explained with a toxicokinetic ion-trapping model. Aquat. Toxicol. 101, 266–275 (2011). 21084122 10.1016/j.aquatox.2010.10.008

[b4] Rendal C., Kusk K. O. & Trapp S. The effect of pH on the uptake and toxicity of the bivalent weak base chloroquine tested on *salix viminalis* and *daphnia magna*. Environ. Toxicol. Chem. 30, 354–359 (2011). 21038438 10.1002/etc.391

[b5] Anskjær G. G., Rendal C. & Kusk K. O. Effect of pH on the toxicity and bioconcentration of sulfadiazine on *Daphnia magna*. Chemosphere 91, 1183–1188 (2013). 23411088 10.1016/j.chemosphere.2013.01.029

[b6] Boström M. L. & Berglund O. Influence of pH-dependent aquatic toxicity of ionizable pharmaceuticals on risk assessments over environmental pH ranges. Water Res. 72, 154–161 (2015). 25262444 10.1016/j.watres.2014.08.040

[b7] Rendal C., Kusk K. O. & Trapp S. Optimal choice of pH for toxicity and bioaccumulation studies of ionizing organic chemicals. Environ. Toxicol. Chem. 30, 2395–2406 (2011). 21823161 10.1002/etc.641

[b8] Schwarzenbach R. P. . The challenge of micropollutants in aquatic systems. Science 313, 1072–1077 (2006). 16931750 10.1126/science.1127291

[b9] Chen Y. . Site-specific water quality criteria for aquatic ecosystems: A case study of pentachlorophenol for Tai Lake, China. Sci. Total Environ. 541, 65–73 (2016). 26398452 10.1016/j.scitotenv.2015.09.006

[b10] Xing L., Liu H., Giesy J. P. & Yu H. pH-dependent aquatic criteria for 2,4-dichlorophenol, 2,4,6-trichlorophenol and pentachlorophenol. Sci. Total Environ. 441, 125–131 (2012). 23137977 10.1016/j.scitotenv.2012.09.060

[b11] Armitage J. M., Arnot J. A., Wania F. & Mackay D. Development and evaluation of a mechanistic bioconcentration model for ionogenic organic chemicals in fish. Environ. Toxicol. Chem. 32, 115–128 (2013). 23023933 10.1002/etc.2020

[b12] Cronin M. T. D., Zhao Y. H. & Yu R. L. pH-dependence and QSAR analysis of the toxicity of phenols and anilines to *Daphnia magna*. Environ. Toxicol. 15, 140–148 (2000).

[b13] Ritchie R. J. & Islam N. Permeability of Methylamine across the Membrane of a Cyanobacterial Cell. New Phytol. 152, 203–211 (2001).

[b14] Escher B. I. & Hermens J. L. M. Internal Exposure: Linking Bioavailability to Effects. Environ. Sci. Technol. 38, 455A–462A (2004). 10.1021/es0406740 15597868

[b15] Ghambarian M., Yamini Y. & Esrafili A. Developments in hollow fiber based liquid-phase microextraction: principles and applications. Microchim Acta 177, 271–294 (2012).

[b16] Watkinson A. J., Murby E. J., Kolpin D. W. & Costanzo S. D. The occurrence of antibiotics in an urban watershed: from wastewater to drinking water. Sci. Total Environ. 407, 2711–2723 (2009). 19138787 10.1016/j.scitotenv.2008.11.059

[b17] Sukul P., Lamshoft M., Zuhlke S. & Spiteller M. Sorption and desorption of sulfadiazine in soil and soil-manure systems. Chemosphere 73, 1344–1350 (2008). 18706672 10.1016/j.chemosphere.2008.06.066

[b18] Wollenberger L., Halling-Sørensen B. & Kusk K. O. Acute and chronic toxicity of veterinary antibiotics to *Daphnia magna*. Chemosphere 40, 723–730 (2000). 10705550 10.1016/s0045-6535(99)00443-9

[b19] De Liguoro M., Fioretto B., Poltronieri C. & Gallina G. The toxicity of sulfamethazine to *Daphnia magna* and its additivity to other veterinary sulfonamides and trimethoprim. Chemosphere 75, 1519–1524 (2009). 19269673 10.1016/j.chemosphere.2009.02.002

[b20] Zhang Y. J., Boyd S. A., Teppen B. J., Tiedje J. M. & Li H. Role of Tetracycline Speciation in the Bioavailability to Escherichia coli for Uptake and Expression of Antibiotic Resistance. Environ. Sci. Technol. 48, 4893–4900 (2014). 24717018 10.1021/es5003428

[b21] Villar-Navarro M., Ramos-Payán M., Fernández-Torres R., Callejón-Mochón M. & Bello-López M. Á. A novel application of three phase hollow fiber based liquid phase microextraction (HF-LPME) for the HPLC determination of two endocrine disrupting compounds (EDCs), n-octylphenol and n-nonylphenol, in environmental waters. Sci. Total Environ. 443, 1–6 (2013). 23178884 10.1016/j.scitotenv.2012.10.071

[b22] Ramos Payán M., López M. Á. B., Fernández-Torres R., Navarro M. V. & Mochón M. C. Hollow fiber-based liquid phase microextraction (HF-LPME) for a highly sensitive HPLC determination of sulfonamides and their main metabolites. J. Chromatogr. B 879, 197–204 (2011). 10.1016/j.jchromb.2010.12.006 21185241

[b23] Tong F. . Hollow-fiber liquid-phase microextraction combined with capillary electrophoresis for trace analysis of sulfonamide compounds. J. Chromatogr. B 942–943, 134–140 (2013). 10.1016/j.jchromb.2013.10.038 24269907

[b24] Han D. & Row K. Trends in liquid-phase microextraction, and its application to environmental and biological samples. Microchim Acta 176, 1–22 (2012).

[b25] Tao Y. . Hollow fiber supported ionic liquid membrane microextraction for determination of sulfonamides in environmental water samples by high-performance liquid chromatography. J. Chromatogr. A 1216, 6259–6266 (2009). 19632683 10.1016/j.chroma.2009.06.025

[b26] Holten Lützhøft H.-C., Vaes W. H. J., Freidig A. P., Halling-Sørensen B. & Hermens J. L. M. Influence of pH and Other Modifying Factors on the Distribution Behavior of 4-Quinolones to Solid Phases and Humic Acids Studied by “Negligible-Depletion” SPME−HPLC. Environ. Sci. Technol. 34, 4989–4994 (2000).

[b27] Chaves A. R., Chiericato Junior G. & Queiroz M. E. Solid-phase microextraction using poly(pyrrole) film and liquid chromatography with UV detection for analysis of antidepressants in plasma samples. Journal of chromatography. B, Analytical technologies in the biomedical and life sciences 877, 587–593 (2009). 19185550 10.1016/j.jchromb.2008.12.070

[b28] Mayer P., Tolls J., Hermens J. L. M. & Mackay D. Equilibrium Sampling Devices. Environ. Sci. Technol. 37, 184A–191A (2003). 10.1021/es032433i 12775037

[b29] Hu X., Liu J., Jonsson J. A. & Jiang G. Development of negligible depletion hollow fiber-protected liquid-phase microextraction for sensing freely dissolved triazines. Environ. Toxicol. Chem. 28, 231–238 (2009). 18937535 10.1897/08-235.1

[b30] Liu J. F., Cai X. Q., Li Z. F. & Jiang G. B. Development of negligible depletion hollow fiber membrane-protected liquid-phase microextraction for simultaneous determination of partitioning coefficients and acid dissociation constants. J. Chromatogr. A 1216, 2583–2586 (2009). 19215929 10.1016/j.chroma.2009.01.092

[b31] Lo I. H. & Hayton W. Effects of pH on the accumulation of sulfonamides by fish. J. Pharmacokinet. Biopharm. 9, 443–459 (1981). 7310643 10.1007/BF01060888

[b32] Nichols J. W. . Observed and modeled effects of pH on bioconcentration of diphenhydramine, a weakly basic pharmaceutical, in fathead minnows. Environ. Toxicol. Chem. 34, 1425–1435 (2015). 25920411 10.1002/etc.2948

[b33] Escher B. I. & Schwarzenbach R. P. Partitioning of Substituted Phenols in Liposome−Water, Biomembrane−Water, and Octanol−Water Systems. Environ. Sci. Technol. 30, 260–270 (1996).

[b34] Xue X., Li L., Chen X., Hu S. & Bai X. Hollow fiber cell fishing with high performance liquid chromatography for screening bioactive compounds from traditional Chinese medicines. J. Chromatogr. A 1280, 75–83 (2013). 23357754 10.1016/j.chroma.2013.01.033

[b35] Liu X., Hu S., Chen X. & Bai X. Hollow fiber cell fishing with high-performance liquid chromatography for rapid screening and analysis of an antitumor-active protoberberine alkaloid group from Coptis chinensis. J. Pharm. Biomed. Anal. 98, 463–475 (2014). 25023388 10.1016/j.jpba.2014.06.030

[b36] Hawthorne S. B., Azzolina N. A., Neuhauser E. F. & Kreitinger J. P. Predicting Bioavailability of sediment polycyclic aromatic hydrocarbons to *Hyalella azteca* using equilibrium partitioning, Supercritical fluid extraction, and pore water concentrations. Environ. Sci. Technol. 41, 6297–6304 (2007). 17937318 10.1021/es0702162

[b37] Fang H., Chu X. Q., Wang X. G., Pang G. H. & Yu Y. L. Using Matrix Solid-Phase Microextraction (Matrix-SPME) to Estimate Bioavailability of DDTs in Soil to Both Earthworm and Vegetables. Arch. Environ. Contam. Toxicol. 58, 62–70 (2010). 19418090 10.1007/s00244-009-9329-4

[b38] Escher B. I. & Hermens J. L. M. Modes of Action in Ecotoxicology: Their Role in Body Burdens, Species Sensitivity, QSARs, and Mixture Effects. Environ. Sci. Technol. 36, 4201–4217 (2002). 12387389 10.1021/es015848h

[b39] Rendal C., Trapp S. & Kusk K. O. Critical evaluation and further development of methods for testing ecotoxicity at multiple pH using *Daphnia magna* and Pseudokirchneriella subcapitata. Environ. Toxicol. Chem. 31, 1843–1852 (2012). 22585467 10.1002/etc.1883

[b40] American Public Health Association (APHA), Standard Methods for the Examination of Water and Wastewaters, 19th ed. American Public Health Association, Washingt on, DC. (1995).

